# Validation of an informatics tool to assess resident’s progress in developing reporting skills

**DOI:** 10.1186/s13244-019-0772-0

**Published:** 2019-09-23

**Authors:** Facundo N. Diaz, Marina Ulla

**Affiliations:** 1 Diagnóstico por Imágenes, Hospital Italiano de Buenos Aires, Juan Domingo Perón 4190, C1199AAB Ciudad Autónoma de Buenos Aires, Argentina; 2 Facultad de Medicina, Universidad de Buenos Aires, II Cátedra de Anatomía, Buenos Aires, Argentina

**Keywords:** Internship and residency, Education, Academic performance, Educational measurement

## Abstract

**Background:**

Diagnostic radiology residency programs pursuits as main objectives of the development of diagnostic capabilities and written communication skills to answer clinicians’ questions of referring clinicians.

There has been also an increasing focus on competencies, rather than just education inputs. Then, to show ongoing professional development is necessary for a system to assess and document resident’s competence in these areas.

Therefore, we propose the implementation of an informatics tool to objectively assess resident’s progress in developing diagnostics and reporting skills. We expect to found decreased preliminary report-final report variability within the course of each year of the residency program.

**Results:**

We analyzed 12,162 evaluations from 32 residents (8 residents per year in a 4-year residency program) in a 7-month period. 73.96% of these evaluations belong to 2nd-year residents.

We chose two indicators to study the evolution of evaluations: the total of discrepancies over the total of preliminary reports (excluding score 0) and the total of likely to be clinically significant discrepancies (scores 2b, 3b, and 4b) over the total of preliminary reports (excluding score 0).

With the analysis of these two indicators over the evaluations of 2nd-year residents, we found a slight decrease in the value of the first indicator and relative stable behavior of the second one.

**Conclusions:**

This tool is useful for objective assessment of reporting skill of radiology residents. It can provide an opportunity for continuing medical education with case-based learning from those cases with clinically significant discrepancies between the preliminary and the final report.

**Electronic supplementary material:**

The online version of this article (10.1186/s13244-019-0772-0) contains supplementary material, which is available to authorized users.

## Key points

- Radiology residency encompasses a wide variety of imaging methods and modalities.
- A radiology training program must have as main objectives for the development of diagnostic capabilities and writing skills to answer clinician’s questions.
- There has been an increasing focus on competencies rather than just education inputs.
- It would be useful to have a tool to assess resident’s progress in developing diagnostics and reporting skills.
- We present our proposal, implementation, and initial results of an informatics tool for objective assessment of reporting skills in radiology residents.

## Background

Diagnostic radiology residency encompasses a variety of diagnostic and image-guided therapeutic techniques including all aspects of image-based diagnosis (radiography, nuclear radiology, diagnostic ultrasound, magnetic resonance, computed tomography, mammography, interventional procedures, and molecular imaging).

The synchronic development of diagnostic capabilities and written communication skills to answer clinician’s questions are the main objectives of a radiologist training program. To show ongoing professional development is necessary for a system to assess and document resident’s competence in these areas.

Therefore, it would be useful to have a tool to assess resident’s progress in developing these diagnostics and reporting skills.

There is seemingly a lack of reference in the literature of a specific and objective tool for this task and how the performance can be measured prospectively.

Thus, our aim is to present the initial results of an informatics tool for objective assessment of the reporting skills of radiology residents.

Our initial expectation was to find a decrease in time on the number the discrepancies between preliminary and final reports and to be able to set a reference for reporting discrepancies (mistakes) of radiology residents.

## Methods

This is a prospective and observational study approved by the ethical committee of our institution.

We work in a 650-bed referral hospital with a 32-resident program, 8 residents per year and 4 years total duration. According to a scale of privileges (or permissions), we expect that every resident went progressively into a four-stage scale from directly supervised report to indirectly supervised report. Regarding reporting skill, 1st-year residents are allowed to observe (level 1) and to perform preliminary reports under direct and on-site supervision (level 2), 2nd- and 3rd-year residents are allowed to perform preliminary reports under immediate available but not on-site supervision (level 3), and 4th-year residents are allowed to perform preliminary reports autonomously with deferred control (level 4).

A large part of the resident training takes place in the consoles of the different modalities (computed tomography, magnetic resonance, fluoroscopy) where the studies are carried out. This is where the resident supervises the work of the radiology technician, applying the study protocol corresponding to the clinical question and interprets the exam.

In this instance, the resident performs a preliminary report of the study in our reporting software. Once the studies are done, they are assigned to the attending radiologist of the different subspecialists, who are responsible to make the final report of the studies, usually the next day. Ultrasound is not included in this study because residents do not perform preliminary ultrasound reports.

The evaluation of reports will be performed at on-duty and moonlight activities, including activities on the reading and emergency rooms. This tool will also create a register of the number and category of preliminary reports, documenting the performance of the residents.

The score for the evaluation will be based on peer review in radiology practice, a well-documented method [1, 2]. Excel 2016 version 16.0 (Microsoft) and R software, version 3.3.1 (R Project for Statistical Computing), were used for statistical analyses.

### Implementing the assessment informatics tool

We made a requirement to the Health Informatics Department of our institution to integrate a button on the reporting software screen that shows a pop-up window for resident evaluation. The attending radiologist prior to signing the report must evaluate the resident’s preliminary report according to a score based on a standardized 5-point rating scale, similar to RADPEER (American College of Radiology) scoring [1]:

(0) No preliminary report;

(1) Concur with interpretation;

(2) Slight discrepancy in interpretation;

(3) Discrepancy by mistake;

(4) Discrepancy by omission

For scores 2 to 4, subheadings are (a) unlikely to be clinically significant and (b) likely to be clinically significant, considering clinically significant discrepancies those findings included in our list of critical findings (potentially life-threatening risk) and those which implicates a change in therapeutic or diagnostic behavior. Despite the 2016 modifications of the RADPEER scoring system to a 3-point scoring system [3], we considered that our modified 5-point scale was more suitable and necessary to the evaluation of radiologist in training, providing a more detailed level of analysis. We also consider the 5-point scale more suitable for Spanish translation. The proposed evaluation score is summarized in Table 1.

**Table 1:** Evaluation score for radiology residents

Score	Description
0	No preliminary report
1	Concur with interpretation
2a	Slight discrepancy in interpretation (unlikely to be clinically significant)
2b	Slight discrepancy in interpretation (likely to be clinically significant)
3a	Discrepancy by mistake in interpretation (unlikely to be clinically significant)
3b	Discrepancy by mistake in interpretation (likely to be clinically significant)
4a	Discrepancy by omission (unlikely to be clinically significant)
4b	Discrepancy by omission (likely to be clinically significant)

Besides, the evaluation score will be a free-text box that allows the evaluator to write any observation or comment that may be relevant for educational purposes. For the implementation of this tool, we decided that evaluation will be an exclusive condition prior to signing the report. Otherwise, the signing button will be not available.

**Fig. 1:**
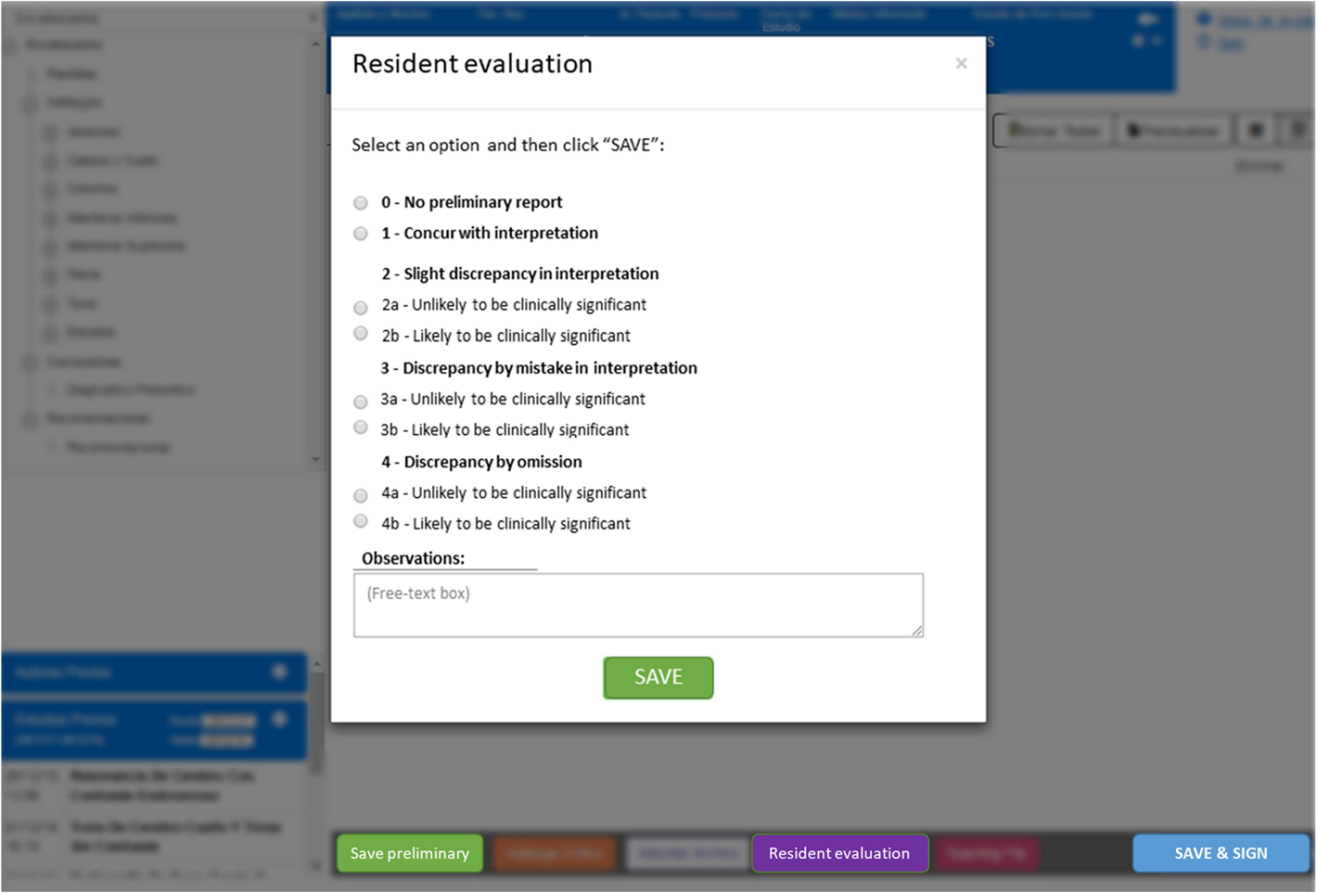
Informatics tool interface. An example of the resident evaluation tool in our reporting software. Workflow: the resident saves the preliminary report (a). The attending radiologist interprets the study, opens the resident evaluation application form (b), and qualifies the preliminary report (c, in this case, concurring with interpretation) in order to finally save and sign the study (d)

An example of the design of the informatics tool interface and the reporting workflow is shown in Fig. 1.

Information about the use of this tool will be provided to the residents and attending radiologists. Through a query to the medical management control board (IBM Cognos Viewer), we will get an Excel table with the following data:

- Name of resident
- Name of attending radiologist
- Data of the preliminary report
- Data of the final report
- ID of patient
- Modality of study (computed tomography, magnetic resonance, radiology)
- Rating scale (0, 1, 2a, 2b, 3a, 3b, 4a, 4b)

The evaluations will be compiled and this will enable us to track the resident’s progress and to give them proper feedback. In keeping with problem-based learning method [4], for cases with the b subheading (clinically significant discrepancies), case-based face-to-face learning sessions will be organized. If it is considered necessary, the referring clinician is called for further information about the case. The resident will prepare the case for discussion under the supervision of the attending radiologist evaluator.

### Three times of analysis of the evaluations will be settled

A weekly analysis, for the detection of the b subheading cases (likely to be clinically significant discrepancies) in order to perform case-based face-to-face learning sessions, in which the resident will prepare the case for discussion under the supervision of the attending radiologist evaluator will be conducted.

An end-of-rotation analysis (every 6 weeks) in order to add objective data from the number of preliminary reports and discrepancies from the final report to the end-of-rotation global assessment will be performed. This type of analysis is especially relevant for subspecialties rotations (neuroradiology, musculoskeletal, body among others).

In addition, there is an on-demand report for those residents that request it for other specific time periods.

## Results

The scoring for preliminary reports of all the 32 residents of our program was included in the analysis. They were randomly analyzed, evaluated, and signed by the attending radiologists of the different subspecialties in their normal daily workflow. The attending can see the name of the reporting resident (in order to solve important issues regarding the patient’s care, it is not an anonymous process).

We excluded from the analysis the scoring of fellowships and those who have a duplicated name on the resident and attending radiologist’s row of the table. The latter could happen because attendings can save a draft version of the report during the reporting process using the “save preliminary report” button, but without any preliminary report by residents.

Basic statistical analysis was performed at first with the whole group and then separated by year of residency.

We requested evaluations between June 1, 2018 and December 31, 2018 obtaining 12,162 (100%) evaluations. We chose this time period according to the 1st semester of the residency program, and the 1st month of the 2nd semester. The details from the general results are shown in Table 2.

**Table 2:** General results of the evaluation of the whole residency program in a 7-month period. *N* = 12,162 evaluations

Evaluation score	Percentage (%)	95% confidence interval (LCI-UCI)
0—No preliminary report	10.24	9.7–10.78
1—Concur with interpretation	74.76	73.99–75.53
2a—Slight discrepancy in interpretation (unlikely to be clinically significant)	6.78	6.33 - 7.23
2b—Slight discrepancy in interpretation (likely to be clinically significant)	3.49	3.16–3.82
3a—Discrepancy by mistake in interpretation (unlikely to be clinically significant)	0.47	0.35–0.59
3b—Discrepancy by mistake in interpretation (likely to be clinically significant)	0.95	0.78–1.12
4a—Discrepancy by omission (unlikely to be clinically significant)	1.70	1.47–1.93
4b—Discrepancy by omission (likely to be clinically significant)	1.60	1.38–1.82

*LCI* lower confidence interval, *UCI* upper confidence interval

On a practical basis, we want to also group the results according to the degree of agreement in concurring (score 1), unlikely to have clinically significant discrepancies (scores 2a, 3a, shown), and likely to have clinically significant discrepancies (scores 2b, 3b, and 4b), shown in Table 3.

**Table 3:** Groups by the degree of agreement

Degree of agreement	Percentage (%)	95% confidence interval (%, LCI-UCI)
Concurring (score 1)	74.76	73.99–75.53
Unlikely to be clinically significant discrepancies (scores 2a, 3a, 4a)	8.95	8.44–9.46
Likely to be clinically significant discrepancies (scores 2b, 3b, 4b)	6.05	5.63–6.47
Not available (score 0)	10.24	9.7–10.78

*LCI* lower confidence interval, *UCI* upper confidence interval

**Fig. 2:**
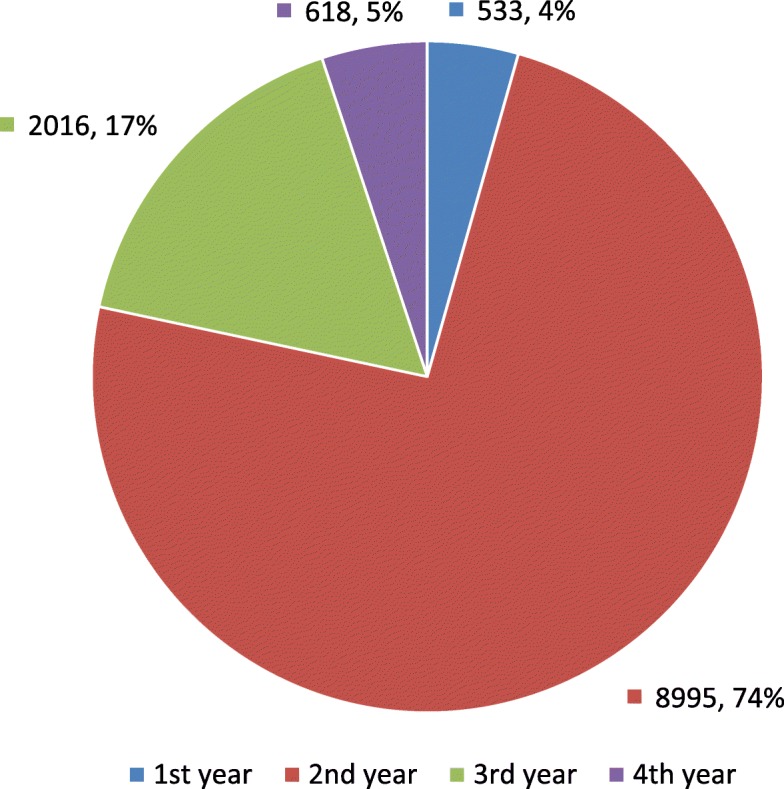
Pie chart showing the distribution of evaluations by year of residency

From this total, 533 (4.38%) evaluations correspond to 1st-year residents, 8995 (73.96%) to 2nd-year residents, 2016 (16.58%) to the 3rd-year, and 618 (5.08%) to the 4th-year (Fig. 2). A detail by year of residency and evaluation score is available in Table 4.

**Table 4:** Distribution by year of residency with evaluation score detail

Year of residency	Total (%)	Evaluation score							
		0	1	2a	2b	3a	3b	4a	4b
1st year	533 (4.38%)	115 (21.58%)	363 (68.11%)	30 (5.63%)	13 (2.44%)	1 (0.19%)	4 (0.75%)	2 (0.38%)	5 (0.94%)
2nd year	8995 (73.96%)	819 (9.11%)	6867 (76.34%)	593 (6.59%)	302 (3.36%)	46 (0.51%)	88 (0.98%)	137 (1.52%)	143 (1.59%)
3rd year	2016 (16.58%)	231 (11.46%)	1430 (70.93%)	141 (6.99%)	85 (4.22%)	7 (0.35%)	20 (0.99%)	59 (2.93%)	43 (2.13%)
4th year	618 (5.08%)	80 (12.94%)	432 (69.90%)	61 (9.87%)	25 (4.05%)	3 (0.49%)	4 (0.65%)	9 (1.46%)	4 (0.65%)
Total	12,162 (100%)	1245 (10.24%)	9092 (74.76%)	825 (6.78%)	425 (3.49%)	57 (0.47%)	116 (0.95%)	207 (1.70%)	195 (1.60%)

The evaluations from 2nd-year residents represent a significant part of the sample (74% of the total). This marked difference is because second-year resident’s night shift hours take place in the emergency CT area, which receives a high flow of patients. First-year resident’s night shift hours take place on CT area too, but without direct responsibility for the preliminary reports. Third-year residents are in MR, and fourth-year residents are in ultrasound.

We performed a detailed monthly analysis of this group (Table 5).

**Table 5:** Distribution by evaluation score over time

Evaluation score	Month							Total
	June	July	August	September	October	November	December	
0	107 (8.47%)	76 (5.34%)	67 (7.14%)	62 (6.38%)	132 (8.95%)	163 (11.07%)	212 (14.63%)	819 (9.11%)
1	960 (75.95%)	1107 (77.79%)	730 (77.74%)	745 (76.65%)	1122 (76.07%)	1144 (77.66%)	1059 (73.08%)	6867 (76.44%)
2a	118 (9.34%)	122 (8.57%)	63 (6.71%)	75 (7.72%)	83 (5.63%)	69 (4.68%)	63 (4.35%)	593 (6.59%)
2b	15 (1.19%)	30 (2.11%)	28 (2.98%)	49 (5.04%)	73 (4.95%)	45 (3.05%)	62 (4.28%)	302 (3.36%)
3a	14 (1.11%)	11 (0.77%)	5 (0.53%)	3 (0.31%)	3 (0.20%)	0 (0.41%)	4 (0.28%)	46 (0.51%)
3b	11 (0.87%)	23 (1.62%)	11 (1.17%)	10 (1.03%)	11 (0.75%)	10 (0.68%)	12 (0.83%)	88 (0.98%)
4a	17 (1.34%)	25 (1.76%)	15 (1.60%)	18 (1.85%)	25 (1.69%)	17 (1.15%)	20 (1.38%)	137 (1.52%)
4b	22 (1.74%)	29 (2.04%)	20 (2.13%)	10 (1.03%)	26 (1.76%)	19 (1.29%)	17 (1.17%)	143 (1.59%)
Total	1264 (14.05%)	1423 (15.82%)	939 (10.44%)	972 (10.81%)	1475 (16.40%)	1473 (16.38%)	1449 (16.11%)	8995 (100%)

**Fig. 3:**
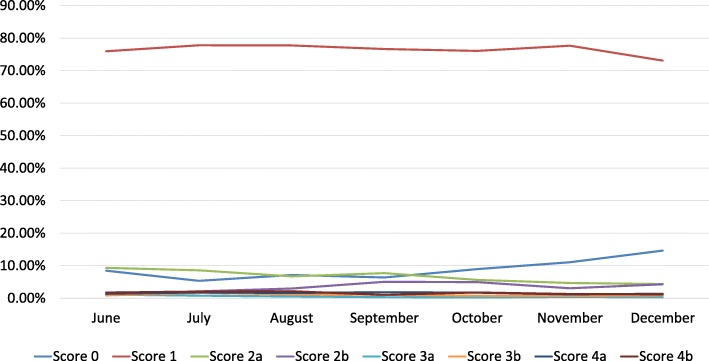
Evolution of scores over time in 2nd-year residents evaluation

A time evolution of scores over time is shown in Fig. 3.

We chose two indicators to evaluate this evolution: the total of discrepancies over the total of preliminary reports (excluding score 0) and the total of likely to be clinically significant discrepancies (scores 2b, 3b, 4b) over the total of preliminary reports (also excluding score 0).

**Fig. 4:**
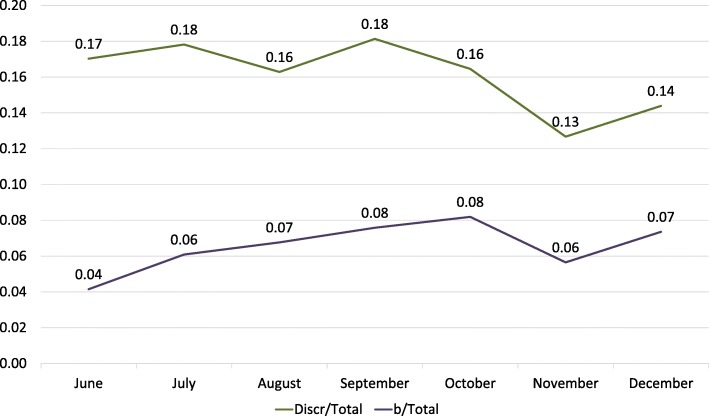
Evolution of indicators over time in 2nd-year residents evaluation. Discr/Total is the total of discrepancies over the total of preliminary reports (excluding score 0), and b/Total is the total of likely to be clinically significant discrepancies (scores 2b, 3b, 4b) over the total of preliminary reports (also excluding score 0)

With the analysis of these two indicators over the evaluations of 2nd-year residents, we found a slight decrease in the value of the first indicator and relative stable behavior of the second one (Fig. 4).

**Fig. 5:**
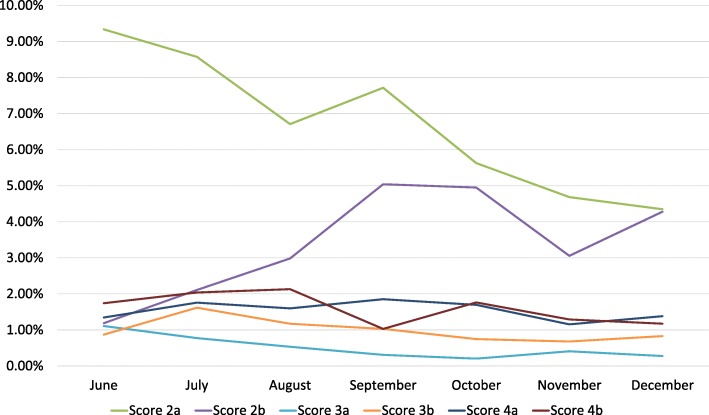
Evolution of indicators over time in the whole group of residents. Discr/total is the total of discrepancies over the total of preliminary reports (excluding score 0), and b/total is the total of likely to be clinically significant discrepancies (scores 2b, 3b, 4b) over the total of preliminary reports (also excluding score 0)

We have found a similar behavior on the analysis of these two indicators in the whole group of residents (Fig. 5).

## Discussion

In recent years within international residency programs, there has been an increasing focus on competencies, rather than just education inputs [5].

While the concept of “knowledge” has been the traditional basis for educational curricula providing a list of topics the trainee is expected to learn, the concepts of skills, competencies, and attitudes are more difficult to appreciate, and in consequence more difficult to assess [5].

The most important background in this field is given by the concepts of competencies, milestones, and entrustable professional activities (EPA) [6].

Competencies refer to the aptitude or suitability to do something or intervene in a certain matter. For example, in the USA, the Accreditation Council for Graduate Medical Education (ACGME) currently define six core competencies: patient care, medical knowledge, practice-based learning and improvement, interpersonal and communication skills, professionalism, and systems-based practice [7]. Each one of them is made up of sub-competencies, and each sub-competency is associated with milestones or goals that residents are required to achieve in order to master the stages of their medical training. In this system, the milestones are usually specialty-specific.

Finally, an EPA is a unit of work or responsibility that attending physicians entrust to the trainee to perform independently once sufficient competency has been demonstrated. This is used as a basis for decisions related to transitioning from residency training to clinical practice [8].

In their work, Deitte et al [8] attempted to establish a framework for the development of EPAs in radiology formulating a list of ten:

- Collaborates as a member of an interprofessional team
- Triages/protocols exams
- Interprets exams and prioritizes a differential diagnosis (reporting skill)
- Communicates results of exams
- Recommends appropriate next steps
- Obtains informed consent and performs procedures
- Manages patients after imaging and procedures
- Formulates clinical questions and retrieves evidence to advance patient care
- Behaves professionally
- Identifies system failures and contributes to a culture of safety and improvement

Image interpretation and diagnosis (EPA-R 3) is among the most fundamental and essential skills that residents acquire during their training, and is linked to radiology milestones as Medical Knowledge 2 (MK2): interpretation of examinations (Table 6).

**Table 6:** Milestone MK2: interpretation of examinations [7]

Level	Objective
1	Makes core observations, formulates differential diagnoses, and recognizes critical findings. Differentiates normal from abnormal
2	Makes secondary observations, narrows the differential diagnosis, and describes management options
3	Provides accurate, focused, and efficient interpretations. Prioritizes differential diagnoses and recommends management
4	Makes subtle observations. Suggests a single diagnosis when appropriate. Integrates current research and literature with guidelines to recommend management
5	Demonstrates expertise and efficiency at a level expected of a subspecialist. Advances the art and science of image interpretation

Until now, in Argentina, there is no published consensus or publications about these educational topics.

In the Diagnostic Radiology Milestone Project [9], they propose the following as possible methods of assessment: end of rotation global assessment, direct observation and feedback, reading out with resident, ER preparedness test, review of reports, rate of major discrepancies, and core exam, each one of them separately requires a lot of time and effort of the attendings and was not possible for every single preliminary report that the resident perform.

Given this context, with focus on the analysis of EPA-R 3: interprets exams and prioritizes a differential diagnosis (reporting skill), we try to find a way to measure the EPA objectively, in order to evaluate the evolution of residents over time.

The design of our tool plays a fundamental role in that it employs informatics data collection and analysis, as well as the integration of daily workflows.

With the implementation of this tool and the discrepancy case-based analysis and presentation, we expected to find decreased preliminary report-final report variability within the course of each year of the residency program and in the 4th-year residency program as a whole (1-year evolution and 4-year evolution).

The 0 score, no preliminary report, deserves a separate analysis. Even though it appears to make some “noise” in the statistical analysis and could be easily excluded, it was helpful for us to detect those cases when the resident omits their obligation of performing a preliminary report. In our residency program, it is mandatory to perform a preliminary report for every study whether it is from emergency or inpatient, and should be completed under resident supervision during duty. Considering that some studies could be too difficult to interpret by the resident on his/her own, and the results of this paper, we decided to establish the desirable level of 0 score below 10%. If some resident exceeds this limit according to the personal control chart, a detailed analysis of the cases in this category will be performed.

The evaluations from 2nd-year residents represent a significant part of the sample (74% of the total) because most of their activities took place in the emergency CT area. Given this fact, added to this evaluation tool, our residency program has an intense training on emergency radiology.

We did not find any significant differences in the evolution of discrepancies over time in residents’ evaluation. Over the 7 months analyzed, there was a slight decline in the total of the discrepancies and no decrease in the rate of probably significant discrepancies (Figs. 4 and 5). We think this is due to the short length of rotations (1.5 months each) which does not allow a long-term follow-up. This implicates a high rate of new exposure to new problems, which implies new possible mistakes. In the other hand, we do not want to extrapolate the comparison between the different years because each group is diverse and we want to see the evolution of each group over the years. We cannot also exclude the possible interpersonal learning ability differences between the residents of each year group.

Also, there were no differences in the rate of significant discrepancies between junior and senior residents (years 1 and 2 and 3 and 4). Junior residents are exposed to the majority of emergency CT and X-ray studies, and senior residents to more complex studies (vascular studies, pneumo-CT [10], virtual colonoscopy [11], spectroscopy, or tractography), so we cannot establish continuity and comparison between these two groups. Considering this “new problems, new mistakes” interpretation, these initial results of the preliminary report evaluations attract the attention of the residency authorities over this issue. We are contemplating to reduce the number of rotations to extend its length, with better follow-up in each area but with the problem of cutting off some of the subspecialties rotations.

We consider that this issue will be a problem in the nearby future, also considering the technical developments and the new modalities like the implementation of artificial intelligence, making it short for a fourth-year residency. Maybe there are too many contents for a 4-year program, and at this point, either we extend the length of the residency by one more year, or translate some of the senior contents to the fellowship programs

According to our results, we decided to establish an upper limit of expected discrepancy rate from preliminary to the final report (or “expected resident error”) at 15% with no more than 7.5% of them likely to be clinically significant discrepancies. This number agrees in part with the reported bibliography regarding disagreements on the retrospective interpretation of studies by the residents [11, 12].

These premises are summarized on Table 7 as proposed standard levels of qualifications for residents (global).

**Table 7:** Proposed standard levels of evaluation score for residency programs

Evaluation score	Desirable level
No preliminary report (score 0)	Below 10%
Concurring with interpretation (score 1)	Above 75%
Discrepancies (scores 2a, 2b, 3a, 3b, 4a, 4b)	Below 15%
Likely to be clinically significant discrepancies (2b, 3b, 4b)	Below 7.5%

There were some previous attempts to measure the diagnostic performance of residents using the unmodified RADPEER scale [13]. Regardless of that, we thought that a modification to the scale was necessary in order to adapt it to an educational residency environment. Our work is the first attempt to objectively measure the reporting skill of radiology residents with an informatics integration in the radiology reporting system and in a daily practice basis. We obtained a large number of evaluations (12,162) that supports our conclusions and proposals.

We strongly believe that this kind of objective assessment tool can be implemented in every residency program worldwide with no major shift, but a combined effort from residency program authorities, radiology residents, and attending radiologists.

Radiology residency programs should consider the use of these reporting assessment tools (or develop some similar tool) in order to quantify and analyze discrepancies between preliminary reports performed by residents and final reports. We provide a printable template version for the evaluation of reporting skill as an additional file (Additional file 1).

We also found that this tool gives objective feedback to trainees after they finish specific rotations, and it creates a register of the number and category of preliminary reports, documenting the performance and procedures of the residents. This system can also be applied to fellowships programs.

Feedback using the observations in the evaluation form and case-based learning sessions for the b subheading (clinically significant discrepancies) is also an important part of the learning process of radiology residents. The importance of feedback in medical education is well-established as it is also well known that case-based learning is an effective learning and teaching method [14, 15].

Finally, we propose that this tool could be included in the Diagnostic Radiology Milestone Project as a possible method of assessment for Milestone MK2: interpretation of examinations.

Our study has some limitations. First, it was conducted in a unique institution. The differences between institutions should not vary substantially, but our results may not be generalizable to all settings.

The ultrasound area has some peculiarities: the ultrasound exam is performed with the final report at the same time by the radiologist. That is, there is no preliminary report instance. The residents have a learning curve wherein in the first year, they accompany a radiologist who is carrying out and reporting, with progressive involvement in ultrasound exam and reporting under the direct supervision of the radiologist through the residency years. To monitor their learning in this area and evaluate them, another methodology has been developed that will be the subject of another publication.

Regardless of the objective intention of the evaluation, there might be some misunderstanding in the interpretation by the attending on the concept of “clinically significant discrepancy.” Periodic reminders and close supervision of the process by the chief resident and program authorities should solve or at least minimize this gap. In an example, is the omission of a hepatic hemangioma in a CT scan with suspicions of acute appendicitis a clinically significant discrepancy or the resident omitted it because it was an emergency-oriented preliminary report? These kinds of issues require consensus in order to be properly evaluated.

Other limitations are evident in the time-consuming task of processing and interpreting this large amount of data. Basic training on data analytics (or a dedicated resource for this task) as well as due time for the analysis should be provided to the supervisors of this tool.

Last but not the least, residents should accept and capitalize on this tool as an educational improvement and set the expectations that the attending radiologist are to fill out the observations field for better feedback. This ultimately benefits them, of course.

As a next step, there should be analyses of longer periods, ideally the 4-year evolution of the same group from the 1st to the 4th year of the residency program with a more detailed segmentation, maybe by rotations, and control charts can offer new insights or new data to be used to improve this tool.

A simplification of the evaluation score could also be considered, such as three categories (concurring, clinically significant discrepancy, and not clinically significant discrepancy). This may be useful for a small number of evaluations. For now, we decided to maintain the original one in order to obtain a detailed classification of the type discrepancy.

Now we have an informatics process established with registered information, and based on it, we can make proper decisions over the residency program.

In conclusion, this tool is useful for objective assessment of the reporting skill of radiology residents. It also opens a channel for better communication between in-training residents and the attending radiologist. Finally, it can provide an opportunity for continuing medical education with case-based learning from those cases with clinically significant discrepancies between the preliminary and the final report.

## Additional file

Additional file 1: Version template for the evaluation of reporting skill of radiology resident. (DOCX 25 kb)

## Data Availability

The data generated and/or analyzed during the current study are not publicly available due to it contains potential sensible data about patients.

## Abbreviations

ACGME: Accreditation Council for Graduate Medical Education
EPA: Entrustable professional activities
MK: Medical Knowledge
