# Supplementary material for: Validation of an informatics tool to assess resident’s progress in developing reporting skills
Source: Insights Imaging. 2019 Sep 23;10:96. doi: 10.1186/s13244-019-0772-0 (PMC6757078; doi:10.1186/s13244-019-0772-0)
Supplement: Supplementary file 1 — Version template for the evaluation of reporting skill of radiology resident. (DOCX 25 kb) [file 13244_2019_772_MOESM1_ESM.docx]

**Additional file: printable version template for the evaluation of reporting skill of radiology resident**

Reporting skill evaluation form

| Name of resident: |  |
| --- | --- |
| Year of residency: |  |
| Rotation: |  |
| Period: |  |

| ID of study | Type | | | Region | Evaluation score | | | | | | | | Observations |
| --- | --- | --- | --- | --- | --- | --- | --- | --- | --- | --- | --- | --- | --- |
|  | Rx | CT | MR |  | 0 | 1 | 2a | 2b | 3a | 3b | 4a | 4b |  |
|  |  |  |  |  |  |  |  |  |  |  |  |  |  |
|  |  |  |  |  |  |  |  |  |  |  |  |  |  |
|  |  |  |  |  |  |  |  |  |  |  |  |  |  |
|  |  |  |  |  |  |  |  |  |  |  |  |  |  |
|  |  |  |  |  |  |  |  |  |  |  |  |  |  |
|  |  |  |  |  |  |  |  |  |  |  |  |  |  |
|  |  |  |  |  |  |  |  |  |  |  |  |  |  |
|  |  |  |  |  |  |  |  |  |  |  |  |  |  |
| ID of study | Type | | | Region | Evaluation score | | | | | | | | Observations |
|  | Rx | CT | MR |  | 0 | 1 | 2a | 2b | 3a | 3b | 4a | 4b |  |
|  |  |  |  |  |  |  |  |  |  |  |  |  |  |
|  |  |  |  |  |  |  |  |  |  |  |  |  |  |
|  |  |  |  |  |  |  |  |  |  |  |  |  |  |
|  |  |  |  |  |  |  |  |  |  |  |  |  |  |
|  |  |  |  |  |  |  |  |  |  |  |  |  |  |
|  |  |  |  |  |  |  |  |  |  |  |  |  |  |
|  |  |  |  |  |  |  |  |  |  |  |  |  |  |
|  |  |  |  |  |  |  |  |  |  |  |  |  |  |
|  |  |  |  |  |  |  |  |  |  |  |  |  |  |
|  |  |  |  |  |  |  |  |  |  |  |  |  |  |
|  |  |  |  |  |  |  |  |  |  |  |  |  |  |
|  |  |  |  |  |  |  |  |  |  |  |  |  |  |
|  |  |  |  |  |  |  |  |  |  |  |  |  |  |
|  |  |  |  |  |  |  |  |  |  |  |  |  |  |
